# Strategies for Developing Functional Secretory Epithelia from Porcine Salivary Gland Explant Outgrowth Culture Models

**DOI:** 10.3390/biom9110657

**Published:** 2019-10-25

**Authors:** Ganokon Urkasemsin, Phoebe Castillo, Sasitorn Rungarunlert, Nuttha Klincumhom, Joao N. Ferreira

**Affiliations:** 1 Department of Preclinical and Applied Animal Science, Faculty of Veterinary Science, Mahidol University, Nakhon Pathom 73170, Thailand; 2 Faculty of Dentistry, National University of Singapore, Singapore 119085, Singapore; 3 Exocrine Gland Biology and Regeneration Research Group, Faculty of Dentistry, Chulalongkorn University, Bangkok 10330, Thailand; 4 Center of Excellence in Regenerative Dentistry, Faculty of Dentistry, Chulalongkorn University, Bangkok 10330, Thailand

**Keywords:** xerostomia, salivary glands, primary cells, progenitor cells, secretory epithelia, neurons, porcine

## Abstract

Research efforts have been made to develop human salivary gland (SG) secretory epithelia for transplantation in patients with SG hypofunction and dry mouth (xerostomia). However, the limited availability of human biopsies hinders the generation of sufficient cell numbers for epithelia formation and regeneration. Porcine SG have several similarities to their human counterparts, hence could replace human cells in SG modelling studies in vitro. Our study aims to establish porcine SG explant outgrowth models to generate functional secretory epithelia for regeneration purposes to rescue hyposalivation. Cells were isolated and expanded from porcine submandibular and parotid gland explants. Flow cytometry, immunocytochemistry, and gene arrays were performed to assess proliferation, standard mesenchymal stem cell, and putative SG epithelial stem/progenitor cell markers. Epithelial differentiation was induced and different SG-specific markers investigated. Functional assays upon neurostimulation determined α-amylase activity, trans-epithelial electrical resistance, and calcium influx. Primary cells exhibited SG epithelial progenitors and proliferation markers. After differentiation, SG markers were abundantly expressed resembling epithelial lineages (E-cadherin, Krt5, Krt14), and myoepithelial (α-smooth muscle actin) and neuronal (β3-tubulin, Chrm3) compartments. Differentiated cells from submandibular gland explant models displayed significantly greater proliferation, number of epithelial progenitors, amylase activity, and epithelial barrier function when compared to parotid gland models. Intracellular calcium was mobilized upon cholinergic and adrenergic neurostimulation. In summary, this study highlights new strategies to develop secretory epithelia from porcine SG explants, suitable for future proof-of-concept SG regeneration studies, as well as for testing novel muscarinic agonists and other biomolecules for dry mouth.

## 1. Introduction

Major salivary glands (SG) such as the submandibular and parotid glands are responsible for the bulk of resting and stimulated saliva secreted, respectively [1,2]. Several systemic maladies, as well as oncological therapies such as radiotherapy for head and neck cancers, can induce hyposalivation and xerostomia [3,4]. The irreversible damage to the innervated secretory epithelium of major SG prompts this hyposalivation phenomenon [5,6,7]. Consequently, these patients suffer from severe oral dryness [2,5,8], with oral tissue repercussions including infections, mucosal inflammation, pain, and tooth loss [9].

Current conventional therapies with saliva cholinergic agonists are not efficacious due to loss of secretory epithelia, particularly in post-radiotherapy patients [4,7,10]. Such functional epithelia loss has led to tissue engineering strategies to model innervated SG epithelia in vitro for the development of future cell transplantation therapies into the injury site [11,12,13,14].

Hence, available and sufficient primary cell sources are needed for such epithelia modelling efforts. Human SG tissue biopsies are either not readily available or found in too limited quantities to generate high numbers of primary cells [15]. Earlier on, researchers usually relied on rodents for the isolation of SG primary cells to overcome this limitation [16,17]. Rodents have advantages since they are inexpensive and readily available, and their SG physiology and histological features are well described. However, their SG anatomical structure and glandular size is less similar to human glands compared with those of larger mammals, including swine [18,19,20,21]. Porcine major SG share similar glandular size, histological, and physiological features with human glands [18,22]. Therefore, swine models can provide sizable amounts of SG tissues, and potentially represent an adequate source of primary epithelial cells (including progenitor/stem cells) towards the generation of bio-engineered models of innervated and functional secretory epithelia. Up to this date, there has been a lack of reports on porcine SG cell culture models for modeling such secretory epithelia [23,24].

The above facts together with the limited availability of large human biopsies/explants from major SG has led us to determine the feasibility of porcine SG explant outgrowth culture models. These porcine models can be utilized to test different regeneration strategies for modelling SG epithelial tissues in vitro. Thus, this study aims to determine whether explant outgrowth culture models from porcine major SGs can be used as a strategy to generate functional epithelia. If these epithelial tissues can provide secretory functions upon neurostimulation, they will be useful to test novel muscarinic agonists in proof-of-concept studies before clinical translation.

## 2. Materials and Methods 

### 2.1. Explant Outgrowth Culture of Porcine SG-Derived Primary Cells

**Figure 1:**
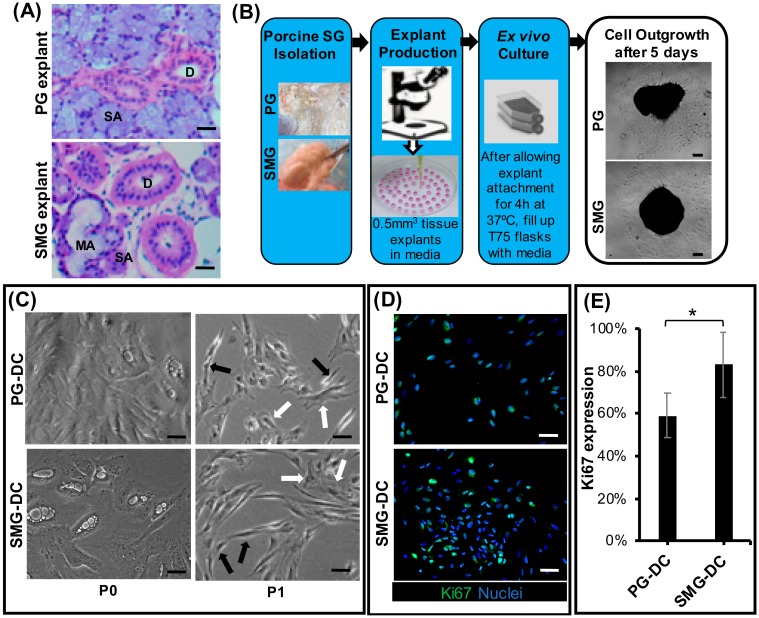
Morphological characterization of parotid- and submandibular-derived cells from ex vivo porcine SG explant outgrowth culture models. (**A**) Submandibular (SMG) and parotid gland (PG) explant tissues via brightfield microscopy after hematoxylin and eosin staining (H&E). Magnification: 20×. Scale bar: 100µm. Legend: SA, serous acini; MA, mucous acini: D, ducts. (**B**) Schematic diagram of the pig explant outgrowth model. (**C**) PG- and SMG-derived cells from the ex vivo model observed via phase-contrast microscopy, showed the presence of heterotypic cell cultures with polygonal (white arrows) and spindle shape (black arrows) cell morphologies after passage 0 (P0). Scale bar: 100 µm. (**D**) Expression of proliferation mitotic marker (Ki67) in PG- and SMG-derived cells after immunofluorescence staining at passage 1. Scale bar: 50 µm. (**E**) Quantification of Ki67+ cell subpopulations in PG-DC and SMG-DC. The y-axis represents protein expression relative to total nuclear/cell counts. *p < 0.001 when comparing SMG-DC with PG-DC. SMG-DC, submandibular gland-derived cells.

This study was performed according to the guidelines set by the National Institute of Health (USA) regarding the care and use of animals for experimental procedures. An explant tissue culture method was utilized to extract and isolate primary cells from porcine major SGs by ex vivo outgrowth. This method is displayed in Figure 1a. Fresh submandibular and parotid glands (SMG or PG, respectively) were retrieved from postmortem 5, 6 month old pigs (*Sus scrofa*), which were available through the comparative medicine animal tissue sharing program and according to the Institutional Animal Care and Use Committee (IACUC) and Internal Review Board at the National University of Singapore (reference numbers/date of approval: 2014-00306/02 September 2014 and B-14-177E/15 September 2015, respectively). All reagents were purchased from Sigma (St. Louis, MO, USA) or Thermo Fisher Scientific (Waltham, MA, USA), unless otherwise indicated. Salivary glands were placed in Hank’s balanced salt solution supplemented with 1% (*v/v*) antibiotic/anti-mycotic and thoroughly washed. Next, glands were freshly dissected using a stereo microscope (Leica Microsystems, Wetzlar, Germany) to remove the connective tissue capsules and septa surrounding the parenchyma, and 0.5 mm^3^ explants were biopsied from the gland’s epithelial parenchyma. Each of these explants was further embedded onto about 200 µl of growth media composed of DMEM (Dulbecco’s Modified Eagle Medium) with GlutaMAX supplemented with 10% (*v/v*) serum and 1% (*v/v*) antibiotic/anti-mycotic. These embedded explants were transferred into cell culture-treated flasks and incubated at 37 °C with 5% CO_2_ (*v/v*) for at least 4 h to promote cell attachment. After incubation, 10 mL of growth media was added with a mycoplasma prophylactic reagent (MycoZap, Lonza, Alpharetta, GA, USA). After 48 h, 10 mL of growth media was added. After 5 days, outgrowth of primary submandibular gland-derived cells (SMG-DC) and parotid gland-derived cells (PG-DC) from the explants could be observed under a brightfield and phase-contrast Leica DMI3000B microscope and images were acquired with a MC120HD camera (Leica Microsystems, Wetzlar, Germany). A mycoplasma detection kit (MycoAlert PLUS, Lonza, Alpharetta, GA, USA) was used through each subculture to monitor for the presence of mollicutes.

### 2.2. Expansion and Differentiation of SMG-DC and PG-DC

Primary cells (SMG-DC and PG-DC) were subcultured after 80% of confluency was reached. Growth media was replaced every 2, 3 days. A cell dissociation reagent (TrypLE) was used to passage according to the manufacturer protocol. The total viable cell count was assessed using the Trypan blue exclusion method at each passage. After such, the population doubling time (PDT) of SMG-DC and PG-DC was calculated as previously described [13]. Subcultures SMG-DC and PG-DC were run up to passage 3. The morphological appearance of SMG-DC and PG-DC was assessed through different passages by acquiring phase-contrast microscopy images at different magnifications (5–20×). Attached cell populations (SMG-DC and PG-DC) from T75 flasks were used for flow cytometry. For gene and protein profiling, 2 × 10^4^ SMG-DC or PG-DC were seeded into 24-well clear flat bottom regular attachment plates. After 80% confluency was reached, either primary cell lysates were produced for gene expression arrays or cells were stained for immunohistochemistry, fluorescence imaging, and protein marker quantification. The growth media for primary cells was changed to a well-established SG differentiation media [13,24] composed of DMEM:F12 supplemented with 1% (*v/v*) antibiotic, 10% (*v/v*) FBS, 20 ng/mL FGF2, 20 ng/mL EGF, 10 ug/mL insulin, N2, and 1 µM dexamethasone. This differentiation stage was run for 7 days, and media was changed every 2, 3 days [13].

### 2.3. Flow Cytometry Analysis

Primary SMG-DC and PG-DC populations were analyzed after the first subculture for cell surface marker expression by flow cytometry. Cells were detached as previously mentioned and re-suspended in MACS buffer (Miltenyi Biotec, Bergisch Gladbach, North Rhine-Westphalia, Germany) at a concentration of 106 per ml. Fluorophore-conjugated antibodies were used to detect standard mesenchymal stem cell (MSC), non-MSC, and putative SG stem/progenitor cell surface markers that are usually present in human and non-human SGs (Table S1). Further, single cell suspensions were incubated with conjugated antibodies for 20 min at 4 °C, after which they were washed with MACS buffer and re-suspended in 500 µL. For each combination of conjugated antibodies, a separate single cell suspension sample was used as a control for incubation with their respective IgG isotype control antibodies. A BD Fortessa (Becton Dickinson, Franklin Lakes, NJ, USA) was employed for pulse-width gate monitoring based detection to analyze the expression of each cell surface marker. A minimum of 105 events per sample was recorded. Flow cytometry was done and analyzed in three biological replicates for each passage using FlowJo version 10 software (Ashland, OR, USA).

### 2.4. Transcriptome Analysis by Quantitative PCR

Quantitative PCR was performed to quantify the mRNA on fresh SMG/PG tissue biopsies/explants and primary cells (SMG-DC, PG-DC). Briefly, we extracted total RNA from biopsied tissues and cells using the Ambion MicroRNA kit following the manufacturer’s protocol. The purity and concentration of extracted total RNA were determined by Nanodrop ND1000 (Thermo Fisher Scientific). Then, complementary DNA (cDNA) was synthesized from total RNA by reverse transcriptase enzyme iScript (Bio-Rad, Hercules, CA, USA) and diluted to 1 ng/µL. Each quantitative polymerase chain reaction (qPCR) was performed according to the manufacturer with the respective cDNA, iTaq solution (Bio-Rad), and optimized forward and reverse oligonucleotide primer sequences in a Bio-Rad CFX96 system. Data was analyzed by a 2-(ddCT) method to calculate relative expression of target genes compared to a house keeping reference gene (S29). All primers were validated in-house by determining their efficiency against serial dilutions of cDNA from fresh porcine SG biopsies. Oligonucleotide sequences of forward and reverse primers are listed in Table S2.

### 2.5. Histological Analysis

For histological and ultrastructural analysis, fresh porcine SMG and PG tissue biopsies/explants (with 0.5 mm thickness) were fixed with 10% (*w/v*) formalin, embedded in paraffin, and subjected to a conventional hematoxylin and eosin staining protocol, published elsewhere [20,25].

### 2.6. Immunofluorescence and Proteome Quantification

To detect pro-mitotic and SG epithelial markers, both fresh SMG and PG tissue biopsies and their respective primary cells were fixed in 4% (*w/v*) paraformaldehyde and an immunohistochemistry protocol was run according to previous publications [14,26]. Fixed SG explant specimens were submerged in a plastic mold filled with optimal cutting temperature (OCT) compound and snap frozen. The frozen specimens were then cryosectioned with a Leica Biosystems CM3050 (Leica, Germany) and 10 µm tissue sections were collected on glass slides. The immunohistochemistry protocol for SG-DC and PG-DC monolayer cells was done in 24-well plates. All explant slides and wells were first permeabilized with 0.1% (*v/v*) Triton X/phosphate buffer saline (PBS), then washed with 0.1% (*v/v*) Tween20/PBS, followed by a blocking step using 10% (*v/v*) donkey serum and 5% (*v/v*) bovine serum albumin for 2 h. This was followed by incubation with primary antibodies overnight at 4 °C, three washing steps with PBS, and 40 min of incubation with secondary antibodies conjugated with fluorophores (Alexa Fluor). Antibodies and their respective dilutions are listed in Table S1. A fluorescent dye to stain the nuclei (Hoechst 33342) was used to counterstain the nuclei. All specimens were imaged and analyzed with a Leica DMi8 fluorescence microscope with a xyz-motorized platform and Leica LAS-X software to obtain a z-stack (scans with 10µm z-steps) in 4 random regions of each culture well. Automated cell counts were done using Image J (version 1.51, NIH, Bethesda, MD, USA) for immuno-fluorescently labeled Ki67+, KRT5+, KRT14+, and αSMA+ cells, and for cells stained with a nuclear fluorescent dye (Hoechst 33342) for each z-stack. Cell counts were normalized to total number of nuclei. Maximum intensity projections from each z-stack were produced.

### 2.7. Western Blot Analysis

To determine the presence of epithelial tight junction proteins in differentiated cells-matrices, immunoblots were run. The preparation of the cell lysates, SDS-PAGE, and Western blot was performed as per standard protocols. Equal loading was verified by detecting total “housekeeping” protein ß-actin, and the final assessment was evaluated with increase concentrations of lysate from SMG-DC differentiated cultures. Cells were harvested after seven days of differentiation in cell lysis buffer containing Pierce RIPA buffer with protease and phosphatase inhibitors. Cell lysates were incubated on ice and homogenized, then centrifuged at 12,000 rpm for 15 min at 4 °C. Cell lysate supernatants were used to measure the protein concentration using Pierce BCA protein assay kit. Protein samples (3–6 µg/lane) were run and separated with Mini-PROTEAN TGX stain-free precast gels (Bio-Rad, Irvine, CA, USA) and transferred to PVDF membranes. Membranes were blocked with 5% (*w/v*) skim milk in 1× tris-buffered Saline with 0.1% Tween 20 (TBST) at room temperature for 1 h and incubated in the primary antibody solution against ZO-1 (1:250; AB59720, Abcam, UK), NKCC (1:1000, ab59791, Abcam, UK), and β-actin (1:2000, A1978, Sigma, USA) at 4 °C overnight. The membranes were incubated with anti-mouse IgG horseradish peroxidase-conjugated secondary antibody in skim milk 1× TBST (1:100, NA934, GE Healthcare, UK) solution. The signal was detected with a Clarity Western ECL kit (Bio-Rad) after several washes with 1× TBST and imaged by chemiluminescence using a ChemiDoc MP Imaging system (Bio-Rad).

### 2.8. Salivary Gland Functional Assays

#### 2.8.1. α-Amylase Enzymatic Activity

To assess the functional secretory enzymatic activity in differentiated cultures, an α-amylase activity screening assay was conducted using an Enzcheck amylase kit (Thermo Fisher Scientific) following the manufacturer’s protocol. Briefly, a standard curve was plotted with serial dilutions of α-amylase. Phenol-free culture media was collected at day 7 from unstimulated and stimulated cultures with 100 µM of Carbachol, a muscarinic agent. Fluorescence intensity was measured by a Tecan M200 multi-well plate reader (Tecan, Männedorf, Switzerland). Final relative fluorescence units (RFU) were calculated by deducting background fluorescence in wells with fresh media only. RFU values were interposed on an amylase standard curve using starch as a substrate to determine final amylase activity.

#### 2.8.2. Epithelial Barrier Function

SMG-DC cells were differentiated for seven days, as per protocol above, in tissue culture hanging inserts with 0.4 µm pore size polyethylene terephthalate membranes (Greiner Bio-one, Kremsmünster, Austria). Epithelial barrier function was assessed via trans-epithelial electrical resistance (TEER) using an EMD Millipore Millicell-ERS2 Volt–Ohm Meter (Thermo Fisher Scientific), as previously described [14]. Final values were calculated by multiplying the total resistance (Ohms) with the effective membrane area (cm^2^) of each insert. TEER measurements can provide an indirect assessment on the presence of epithelial tight junction proteins and the epithelial barrier function. Undifferentiated cells (SMG-DC) in transwell filters were used as a negative control.

#### 2.8.3. Intracellular Calcium Activity

To measure the intracellular calcium influx in differentiated primary cell cultures (after seven days of differentiation), a Fluo-4 Direct Calcium Assay kit (Thermo Fisher Scientific) was used according to the manufacturer instructions. Calcium chloride was first added to cell-matrix cultures. Next, cultures were stimulated with either Carbachol or Isoproterenol. In preliminary studies, differentiated cells were stimulated with increasing concentrations of Carbachol (10–1000 µM) and Isoproterenol (1–100 µM) to optimize the concentrations of such neurotransmitters in this new porcine primary cell culture system. The calcium fluorescence intensity was visualized and quantified as described previously [14]. All values were normalized to total nuclei present. After staining, calcium intracellular influx was visualized by time-lapse imaging as set by the LAS X software on a Leica DMi8 fluorescence microscope, as reported above. Sequential image acquisition was performed every 5 s per cycle before and after neurostimulation.

### 2.9. Statistical Analysis

All data were normally distributed. For flow cytometry and PCR, the data is displayed as mean ± standard error of the mean from three independent groups/culture wells. For the remaining data, plotted column bars represent mean and error bars standard deviation (SD) from four to eight independent culture wells. Paired Student’s *t*-tests were performed for time-dependent experiments to determine the consistency of PDT through different passages for both PG-DC and SMG-DC. Unpaired Student t-tests were performed to compare between two groups, as follows: PG-DC versus SMG-DC; native SG biopsies versus passage 1; undifferentiated versus differentiated PG-DC/SMG-DC, unstimulated versus stimulated (with Carbachol or Isoproterenol). Statistical significance was set at *p* < 0.05. All statistical analysis was conducted using Graphpad Prism version 7 software (Graphpad Software Inc., San Diego, CA, USA).

## 3. Results

### 3.1. Porcine PG and SMG Primary Cells Had Heterotypic Morphology

After removal of the connective tissue capsules, SMG and PG tissue explants exhibited similar acinar and ductal parenchyma morphologies similar to their human counterparts (Figure 1A and Figure S1). Porcine explant outgrowth cultures from PG and SMG showed a consistent cellular outgrowth after five days (Figure 1B). Primary cells from PG/SMG explant cultures exhibited a polygonal epithelial-like form and a spindle shape mesenchymal-like morphology (Figure 1C). These observations indicated these cultures are heterotypic and may possess the potential to recapitulate, in vitro, the epithelial secretory compartments found in the functional SG. In addition, the majority of primary cells at first subculture possessed a pro-mitotic proliferative activity shown after Ki67 immunostaining (Figure 1D, E). Ki67 is a well-known transcription factor and a key regulator of the mitotic cycle. However, SMG-DC had the highest number of Ki67+ cells (Figure 1E). A low population doubling time (PDT), ranging from 43 to 85 h, was noted at earlier passages (Figure S2). This may indicate a high proliferation and up-scalability potential, important towards our goal of generating functional epithelial secretory tissues in vitro. These findings led to the selection of early subcultures (passage 1) of PG-DC and SMG-DC for subsequent experiments.

### 3.2. Primary Cell Subcultures Contained Large Putative SG Epithelial Stem/Progenitor Cell Subpopulations

**Figure 2:**
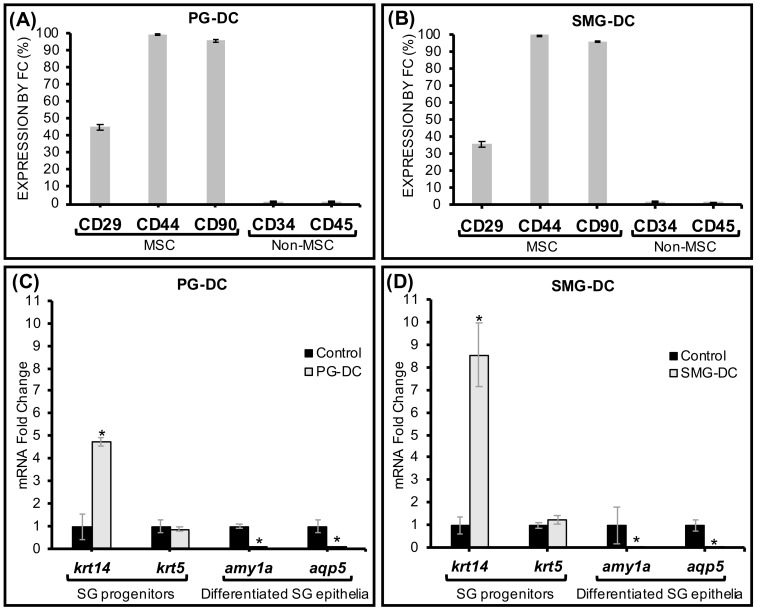
PG-DC and SMG-DC mainly expressed standard MSC markers and SG epithelial progenitor markers in their proteome and transcriptome. (**A**,**B**) Proteomic expression of mesenchymal stem cell (MSC) and putative SG stem/progenitor cell surface markers (CD29, CD44, CD90) and non-MSC surface markers (CD34, CD45) by flow cytometry (FC) after first subcultures, *N* = 3, 4. The expression of other cell surface stem cell markers is displayed in Table S3. (**C**,**D**) mRNA expression by qPCR of undifferentiated and differentiated SG epithelial markers in cells from first passage is represented in the Y-axis as fold change relative to controls (which are mRNA samples from either SMG or PG fresh explant tissues) and normalized to a house keeping gene (*S29*). *p < 0.05 when compared to control, *N* = 3, 4. PG-DC, parotid gland-derived cells; SMG-DC, submandibular gland-derived cells.

The presence of surface markers such as CD29, CD44, and CD90 is commonly used as a criterion to identify human MSCs [27]. Approximately 33–50% of the PG-DC and SMG-DC expressed CD29 (Figure 2A,B), which is also broadly present in human fetal and adult SG epithelial and myoepithelial cells [28]. CD29 is also a putative SG epithelial stem/progenitor cell marker capable of undergoing epithelial differentiation and inducing SG regeneration when transplanted in vivo [29]. Moreover, more than 90% of SMG-DC and PG-DC were CD44+ and CD90+ (Figure 2A,B). These latter surface markers are found in human SG-derived multipotent MSC in both major [30,31] and minor glands [32]. As expected, non-MSC markers (CD34, CD45) were scant in SMG-DC and PG-DC cultures (Figure 2A,B), alike human SGs [32]. At the transcriptome level, CD29 and CD90 were significantly upregulated after cell isolation (Table S3), supporting the flow cytometry findings for these same markers.

### 3.3. Primary Cell Subcultures Had Up-Regulated Genes Associated with SG Epithelial Progenitors

Transcriptome analysis by PCR showed either a maintenance or upregulation in the expression of genes in first subcultures (relative to SG native tissues) that are specific to subpopulations of epithelial ductal progenitors like krt5 (cytokeratin 5) and krt14 (cytokeratin 14) in both PG-DC and SMG-DC (Figure 2C,D). The krt14 marker was found expressed in self-duplicating SG ductal epithelial cells with high turnover properties [33,34]. Therefore, this enrichment of krt14 mRNA indicated the presence of proliferative cells in PG-DC and SMG-DC with high expansion capacity. Moreover, in early subcultures there were no significant changes in the expression of other putative acinar and ductal epithelial progenitor genes, kit and krt19, respectively (Table S3). Genetic markers specific to mature secretory acinar cells were down-regulated (amylase—*amy1a*, aquaporin 5—*aqp5*) at first subcultures (Figure 2C,D).

Overall, these observations at the transcriptome level suggest that PG-DC and SMG-DC have an undifferentiated epithelial-like genetic background. In proteomic studies using immunofluorescence imaging, more than 50% of PG-DC and SMG-DC abundantly expressed Ki67, which largely overlapped with cytokeratin 14-positive cells (KRT14+), suggesting a high pro-mitotic activity and proliferation ability of the undifferentiated epithelial KRT14+ populations (Figure S2B,C). These proteome data confirmed our transcriptome findings, and also revealed that SMG-DC have significantly more KRT14+ progenitors, as well as proliferative cells, than PG-DC.

### 3.4. Secretory Epithelial and Myoepithelial Cells Were Abundant in Differentiated SMG-DC and PG-DC

**Figure 3:**
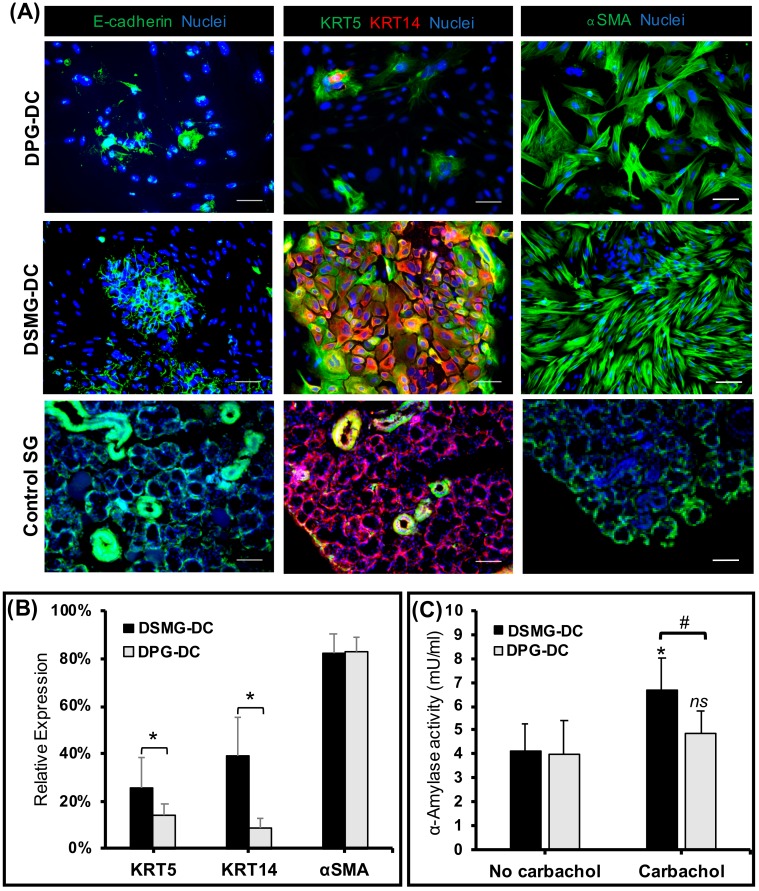
Expression of SG-specific epithelial and myoepithelial cell markers and amylase activity was mainly notable in differentiated SMG-DC (DSMG-DC). (**A**) Fluorescence microscopy images with z-stack maximum intensity projections are shown from immunofluorescence for E-cadherin, KRT5, KRT14, and αSMA epithelial markers and counterstained with a nuclear dye. DPG-DC and DSMG-DC micrographs are at 20× magnification—scale bar: 100 µm. Control SG group are at 10× magnification and represent fresh porcine SG explant tissue where all SG markers should be expressed—scale bar: 200 µm. (**B**) Immunofluorescence quantification of SG progenitor (KRT5+ and KRT14+) and myoepithelial (αSMA) cell subpopulations in DSMG-DC and DPG-DC. The y-axis represents protein expression relative to total nuclear/cell counts. *p < 0.05 when comparing DSMG-DC with DPG-DC. (**C**) SG amylase activity in DSMG-DC and DPG-DC before and after cholinergic stimulation (Carbachol 100 µM). * *p* < 0.05 and *ns-*not significant when comparing unstimulated (no carbachol) with cholinergic stimulation. # *p* < 0.05 when comparing DSMG-DC with DPG-DC. DPG-DC, differentiated PG-DC; DSMG-DC, differentiated SMG-DC.

After SG epithelial differentiation, we assessed the expression of SG-specific epithelial (acinar and ductal) and myoepithelial proteins by immunofluorescence (Figure 3A,B). Both differentiated SMG-DC and PG-DC (labeled as DSMG-DC and DPG-DC, respectively) abundantly possessed KRT5, KRT14, e-cadherin, and alpha-smooth muscle actin (αSMA) proteins, indicating the presence of epithelial and myoepithelial cell lineages. Despite this, DSMG-DC had a greater number of epithelial ductal progenitors (KRT5- and KRT14-positive immunostained cells) enclosed in epithelial cell adherens junctions (E-cadherin-positive cells) as compared to DPG-DC. However, high numbers of myoepithelial cells (αSMA-positive) were found in both differentiated cultures (Figure 3B). During development, subpopulations of myoepithelial cells can co-express epithelial progenitor markers [33,34].

### 3.5. DSMG-DC Had Greater SG-Like Functional Properties

**Figure 4:**
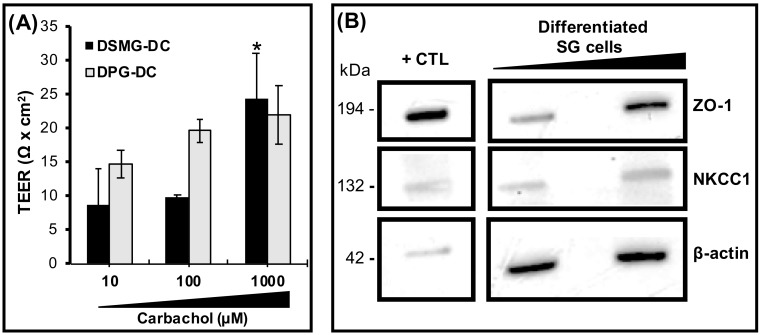
Epithelial barrier function in differentiated SG cell cultures. (**A**) Epithelial permeability of SG differentiated cells was assessed by trans-epithelial electrical resistance (TEER) upon stimulation with increasing concentrations of Carbachol. *p < 0.05 when comparing with lower concentrations. (**B**) Expression of epithelial tight junctions and water channel proteins while loading different protein concentrations (3 and 6 µg) of differentiated SG cells from passage 1 via Western blot. Loading control and “housekeeping” protein was β-actin and positive control tissue (+CTL) was from fresh SMG biopsy.

To evaluate the secretory function of DPG-DC/DSMG-DC subcultures, the salivary enzyme α-amylase was assessed and found significantly increased in DSMG-DC after cholinergic stimulation with an acetylcholine analogue, Carbachol (Figure 3C). Differentiated porcine subcultures were tested for epithelial barrier function and functional innervation. These cells exhibited an increased trans-epithelial electrical resistance (TEER) compared to DPG-DC (Figure 4A) upon cholinergic stimulation. This epithelial barrier function was supported by the presence of SG epithelial tight junction proteins, such as ZO-1 (Figure 4B). Other differentiated SG secretory epithelial markers (ZO-1, NKCC1) as well as a SG basement membrane proteoglycans (Perlecan) were also present in differentiated SG cell cultures and respective extracellular matrices (Figure S3 and Figure 4B). This suggests the potential of these differentiated cell cultures to achieve epithelial integrity and barrier function during neurostimulation. However, functional water channels such as Aquaporin 1 (AQP1), a ductal epithelial marker, could not be clearly located on the apical plasma membrane (Figure S4).

### 3.6. Differentiated SG Primary Cells Displayed Responses to Neurotransmitters

**Figure 5:**
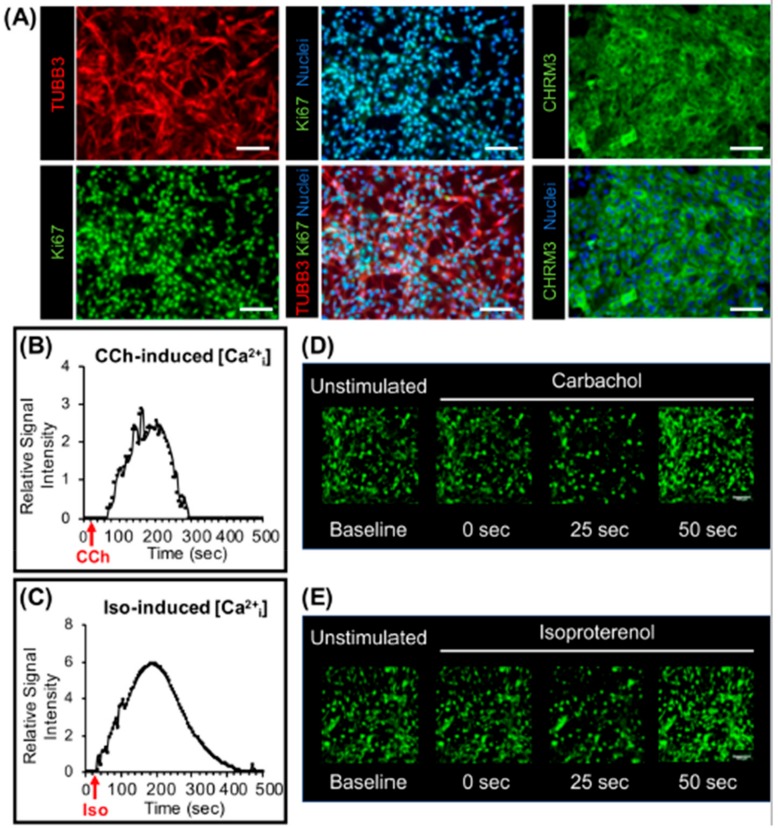
Neuronal subpopulations and intracellular Ca^2+^ influx in differentiated SG cells upon cholinergic and adrenergic stimulation. (**A**) Innervation determined by immunofluorescence with β3-tubulin (TUBB3), Cholinergic receptor muscarinic 3 (CHRM3), and Ki67 markers, and counterstained with a nuclear dye. Scale bar: 100 µm. (**B**,**C**) Intracellular calcium influx in differentiated SG cells after (**B**) cholinergic stimulation with Carbachol (CCh) 10μM and (**C**) adrenergic stimulation with Isoproterenol (Iso) 10μM at cycle 5. Data are representative of 3 biological replicates and are presented as a relative signal intensity where each reading was normalized to the baseline calcium influx (at unstimulated state). The remaining replicates are displayed in Figure S5. (**D**,**E**) Representative microscopy real time-lapsed images showing calcium ion [Ca^2+^i] mobilization in differentiated cell cultures after calcium fluorescence labeling (in green), before and after: (**D**) Cholinergic stimulation with CCh and (**E**) adrenergic stimulation with Iso. Mag.: 20×.

Due to their prominent α-amylase secretory and barrier function, viability (a significantly higher number of Ki67-positive cells) and epithelial enrichment (higher KRT5 and KRT14 expression), DSMG-DC were evaluated for neuronal markers and cellular responses were recorded upon neurostimulation. These differentiated SG subcultures were immunostained for the pan-neuronal marker β3-tubulin (TUBB3) and cholinergic receptor muscarinic 3 (CHRM3) and cultures exhibited a profuse and viable neuronal network as well as abundant CHRM3-positive cells susceptible to neurostimulation (Figure 5A). To assess whether differentiated cells can be stimulated by neurotransmitters, intracellular calcium influx was evaluated upon supplementing the media with cholinergic and adrenergic agonists (Carbachol and Isoproterenol, respectively). Calcium influx increased in cell cultures after neurostimulation with both neurotransmitters (Figure 5B–E).

Taken together, the proteomic and functional characterization data indicate that porcine major SG primary cells from explant outgrowth models can form a functional secretory epithelium after in vitro differentiation.

## 4. Discussion

Herein, an explant outgrowth culture model using porcine major SG explants could, for the first time, successfully generate an enriched epithelial progenitor population and differentiate them into neurons and functional SG-like epithelial secretory cultures in two-dimensional (2D) microenvironments. These cultures were heterotypic in nature as they were composed of acinar and ductal epithelial cells as well as myoepithelial cells and neurons. Other explant ex vivo models have been tested for other exocrine glands including mammary [35] and sebaceous glands [36], and shown to produce and maintain appropriate homeostatic conditions for proliferative epithelial populations.

A few studies have reported on the generation of 2D functional epithelial cultures in vitro from ex vivo explant outgrowth of human minor SG [32,37], though not from human major SG. These latter studies did not directly assess the present of innervation. Our group has recently developed innervated epithelial organoids from human cells [14]. However, the poor availability of human cells from healthy donors has led us to seek new methodologies using porcine SG explants and their respective primary cells for modelling the SG in vitro. As previously reported, porcine SG have several similarities to their human counterparts in terms of morphology, histology, architecture, and physiology [18,22,38], leading researchers to use in vivo miniature porcine models [25,38,39,40] and a ductal ligation injury model [41].

In our explant culture model with porcine major SG, we produced primary cell subcultures that could be expanded up to passage 3 with a doubling time between 2 and 3 days. Furthermore, these subcultures were proliferative (pro-mitotic) and heterotypic in morphology. The majority of the Ki67+ proliferative cells were epithelial ductal progenitors (KRT14+) and expressed putative epithelial and mesenchymal stem cell markers (e.g., CD29, CD44, CD90). The study by Lu and colleagues [32] using human minor SG explants also developed comparable proliferative populations. Jang and colleagues [37] have used a comparable ex vivo explant culture with the same type of glands, but in combination with collagen matrices and several xeno-derived growth factors to generate functional acinar-like cells. These minor SG are easily assessible and retrieved from the oral cavity, however, they have limited epithelial acinar/ductal cellular compartment, which produces less than 10% for the total saliva produced in humans [42].

Recently, in human fetal and adult SG, Togarrati and others [28] observed the wide expression of CD29 in several cellular compartments, including acinar and ductal epithelial, myoepithelial, and mesenchymal stromal cells. Same authors have proposed the use of such CD29+ cells to devise new cellular therapies for SG disorders. In our study, porcine explants from major SG were also found to generate a large number of CD29 expressing cells (~50%) and such enriched cell populations formed acinar and ductal epithelial and myoepithelial cultures after differentiation in vitro. Although, other relevant studies have enriched human epithelial SG cultures for translational applications without antigenic sorting and using serum free conditions [11].

More importantly, a profuse neuronal network was also present in these heterotypic epithelial cultures. Differentiated cultures derived from submandibular gland explants exhibited SG-like secretory functions upon cholinergic stimulation with an increased amylase secretion and intracellular calcium activity. Previous SG explant culture reports have not determined the presence of a neuronal network in human- and mouse-derived primary salivary epithelial cell cultures [17,32,37]. On the other hand, in 2D co-culture systems, one report has shown that neurons self-organize when culturing rat salivary epithelial cells with mouse cortical neurons [43]. Innervation of epithelial salivary gland-like cultures using different types of human-derived cells has been difficult to achieve and our group has overcome this limitation recently in novel 3D culture systems [14,24]. In addition, maintenance of neuronal phenotypes and preservation of their functional homeostasis for long periods of time (through passaging and culture) remains to be verified in 2D and 3D systems, and should be explored in future studies [44]. Moreover, 3D salivary cultures allow researchers to surpass limitations present in 2D microenvironments, including the lack of multi-lumen formation, difficulties in evaluating tubulogenesis, and complexities from overall experimental techniques to characterize SG microscopic, structural, and biochemical features usually found in vivo [43,45]. However, proteome analysis on porcine cells/tissues and regenerative therapy models remains challenging due to the limited number of commercially available antibodies with specific porcine immunoreactivity. Thus, based on the above premises, it was challenging to locate functional water channels (AQP1) on the apical membrane of epithelial cells to determine the epithelial polarity in our 2D culture models of porcine SG-derived cells.

Moreover, vascularization is yet to be demonstrated in both 2D and 3D SG culture systems. Future studies may investigate angiogenesis in primary cell cultures from porcine SG explants upon the addition of signaling cues (e.g., vascular endothelial growth factor) or with other cell lines in co-culture (e.g., endothelial cells). Post-radiotherapy injury to the porcine SG primary cells will be explored in the future to better design cell-based therapies to ameliorate such injuries. However, for the purpose of SG epithelia transplantation, translational studies with human SG cells are essential to achieve biocompatibility [11]. Furthermore, this explant outgrowth culture system can also supply primary cells for modelling the SG in vitro for proof-of-concept studies to evaluate our current [14,24] and future regeneration strategies.

## 5. Conclusions

In summary, this porcine SG explant culture strategy can generate populations of epithelial progenitors, as well as differentiated epithelial (acinar, ductal, myoepithelial) and neuronal cells, leading to a heterotypic epithelia. In addition, this epithelia provided secretory functions upon cholinergic and adrenergic stimulation with established neurotransmitters. Thus, the developed secretory epithelia can be used for in vitro screening of novel muscarinic drugs conceived to treat hyposalivation.

## Supplementary Materials

The following are available online at https://www.mdpi.com/2218-273X/9/11/657/s1, Figure S1: Human major salivary glands share very similar histological features and glandular sizes with the porcine glands, but not with the mouse submandibular glands.; Figure S2: Cellular expansion through passaging and proliferation of epithelial progenitors in non-confluent undifferentiated SMG-DC and PG-DC cultures; Figure S3: Expression of SG-specific acinar and ductal epithelial, myoepithelial and basement membrane markers after SG differentiation; Figure S4: AQP1 water channels were not precisely located at the apical membrane after SG differentiation; Figure S5: Intracellular Ca2+ influx tracking in differentiated SG cells upon cholinergic and adrenergic stimulation. Table S1: List of primary antibodies (conjugated and unconjugated) used for flow cytometry or immunofluorescence imaging; Table S2: Oligonucleotide forward and reverse primer sequences optimized for porcine SG tissues; Table S3: Expression of other mesenchymal and SG transcriptome markers in SMG-DC and PG-DC.
