# Supplementary material for: Strategies for Developing Functional Secretory Epithelia from Porcine Salivary Gland Explant Outgrowth Culture Models
Source: Biomolecules. 2019 Oct 25;9(11):657. doi: 10.3390/biom9110657 (PMC6921070; doi:10.3390/biom9110657)
Supplement: Supplementary file 1 [file biomolecules-09-00657-s001.pdf]

biomolecules

*Supplementary Materials*

# Strategies for Developing Functional Secretory Epithelia from Porcine Salivary Gland Explant Outgrowth Culture Models

**Ganokon Urkasemsin [1], Phoebe Castillo [2], Sasitorn Rungarunlert [1], Nuttha Klincumhom [3,4] and Joao N. Ferreira [3,4,*]**

[1] Department of Preclinical and Applied Animal Science, Faculty of Veterinary Science, Mahidol University, Nakhon Pathom, 73170, Thailand
[2] Faculty of Dentistry, National University of Singapore, 119085, Singapore
[3] Exocrine Gland Biology and Regeneration Research Group, Faculty of Dentistry, Chulalongkorn University, Bangkok, 10330, Thailand
[4] Center of Excellence in Regenerative Dentistry, Faculty of Dentistry, Chulalongkorn University, Bangkok, 10330, Thailand
* Correspondence: Joao.F@chula.ac.th; Tel.: +6622188816; Fax: +6622188810.

**Table S1.** List of primary antibodies (conjugated and unconjugated) used for flow cytometry or immunofluorescence imaging. Manufacturers location: Biolegend, San Diego, CA, USA; Abcam, Cambridge, UK; Cell Signaling Technology, Danvers, MA, USA; Novus Biologicals, Centennial, CO USA; R&D systems, Minneapolis, MN, USA; Santa Cruz, Dallas, TX, USA; Biorbyt, Cambridge, UK; Merck, Darmstadt, Germany.

| Antibody | Dilution used | Catalog No. | Manufacturer |
|---|---|---|---|
| Alexa Fluor 488 anti-CD29 | 1:300 | 303015 | Biolegend |
| Alexa Fluor 647 anti-CD44 | 1:200 | 103017 | Biolegend |
| PE anti-CD90 | 1:80 | 328109 | Biolegend |
| Alexa Fluor 647 anti-CD34 | 1:300 | 343507 | Biolegend |
| Pacific Blue anti-CD45 | 1:200 | 304021 | Biolegend |
| anti-KRT5 | 1:200 | AB24647 | Abcam |
| anti-KRT14 | 1:200 | 181595 | Abcam |
| anti-E-cadherin | 1:100 | 3195 | Cell Signaling Technology |
| anti-Ki67 | 1:100 | AF7617 | Novus Biologicals |
| anti-αSMA | 1:200 | 5694 | Abcam |
| anti-beta-III tubulin | 1:200 | MAB1195 | R&D systems |
| anti-Chrm3/M3 | 1:200 | SC-9108 | Santa Cruz |
| anti-Aquaporin 1 | 1:50 | orb10122 | Biorbyt |
| anti-Perlecan | 1:500 | MAB1948P | Merck |

**Table S2.** Oligonucleotide forward and reverse primer sequences optimized for porcine SG tissues.

| Gene | Forward sequence | Reverse sequence | NCBI reference |
|---|---|---|---|
| *CD29* | TGCAACCCCAACTACACTG | TTACAGACCCCACATTCACAG | NM_213968 |
| *CD90* | TCCAGCAAGTACGACATCAAG | GAGAGGTGGAGTTCGCATG | NM_001146129 |
| *krt5* | CGTAGTCAATCAAGCCTATT | GAAGCAGAGCGTAGAATC | XM_003126173 |
| *krt14* | GAGATGTGACCTCCACTC | TAGTTCTTGGTGCGAAGG | XM_021067000 |
| *krt19* | TCAGTGTGGAGGTTGATTCG | CTCATACTGGCTTCTCATGTCG | XM_003131437 |
| *kit* | CGGAGAGCATTCAGACTTA | GCATCATCATCATCATATTGTTAC | NM_001044525 |
| *amy1a* | AGCCCTTGTCTTTGTTGATA | ACTCGTGTGAATCCGTAAG | XM_021090124 |
| *aqp5* | ATGATTCTGACCTTCCAGCTG | GTGAAGTAGATCCCCACAAGG | NM_001110424 |

**Table S3.** Expression of other mesenchymal and SG transcriptome markers in SMG-DC and PG-DC. mRNA expression from PCR is represented as fold change in cells from P1 relative to fresh SG tissue biopsies and normalized to a house keeping gene (S29). F.C.: fold change. SEM: standard error of the mean. * indicates $p < 0.05$ between SMG-DC passage 1 cells and fresh SMG tissue biopsies (baseline group). ** indicates $p < 0.05$ between PG-DC passage 1 cells and fresh PG tissue biopsies (baseline group).

| SMG-DC | | |
|---|---|---|
| **Gene** | **Mean F.C.** | **SEM** |
| *CD29* | 52.27* | 18.56 |
| *CD90* | 7.62* | 2.90 |
| *krt5* | 1.25 | 0.20 |
| *kit* | 0.07 | 0.04 |
| *krt14* | 8.55* | 1.39 |
| *krt19* | 0.80 | 1.03 |
| *amy1a* | 0.06* | 0.01 |
| *aqp5* | 0.02* | 0.01 |
| **PG-DC** | | |
| **Gene** | **Mean F.C.** | **SEM** |
| *CD29* | 37.24** | 7.17 |
| *CD90* | 10.31** | 1.76 |
| *krt5* | 0.86** | 0.08 |
| *kit* | 0.03 | 0.01 |
| *krt14* | 4.74** | 0.21 |
| *krt19* | 2.65 | 0.09 |
| *amy1a* | <0.01** | <0.001 |
| *aqp5* | 0.02** | 0.01 |

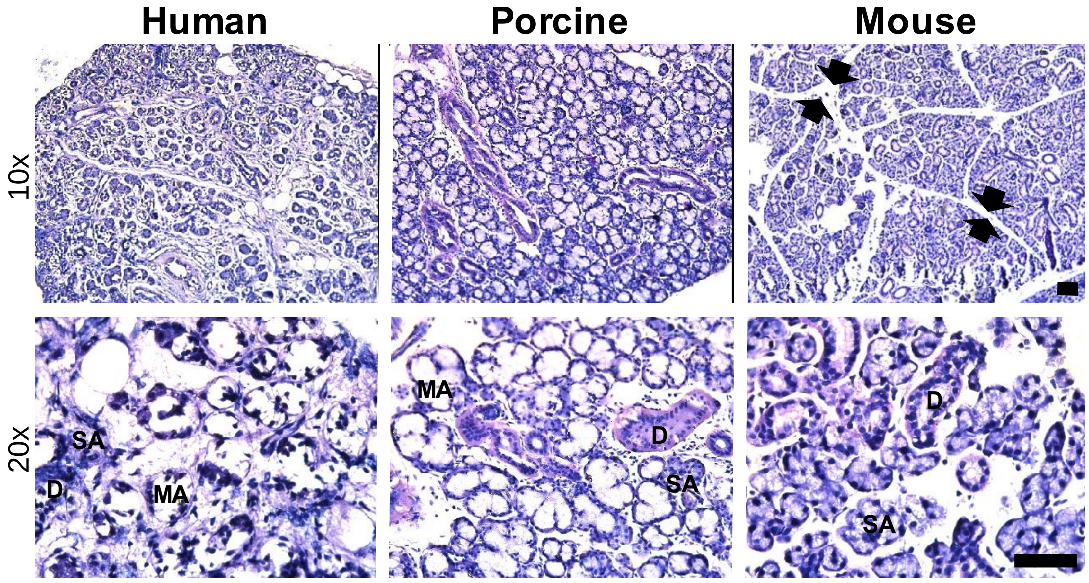


**Figure S1.** Human major salivary glands share very similar histological features and glandular sizes with the porcine glands, but not with the mouse submandibular glands. Mouse glands have a prominent connective tissue capsule (black arrows) surrounding all acinar lobes, and they lack mucous acinar cells. Hematoxylin and eosin staining. Scale bar: 100 µm. Legend - SA: serous acini. MA: mucous acini. D: Ducts.

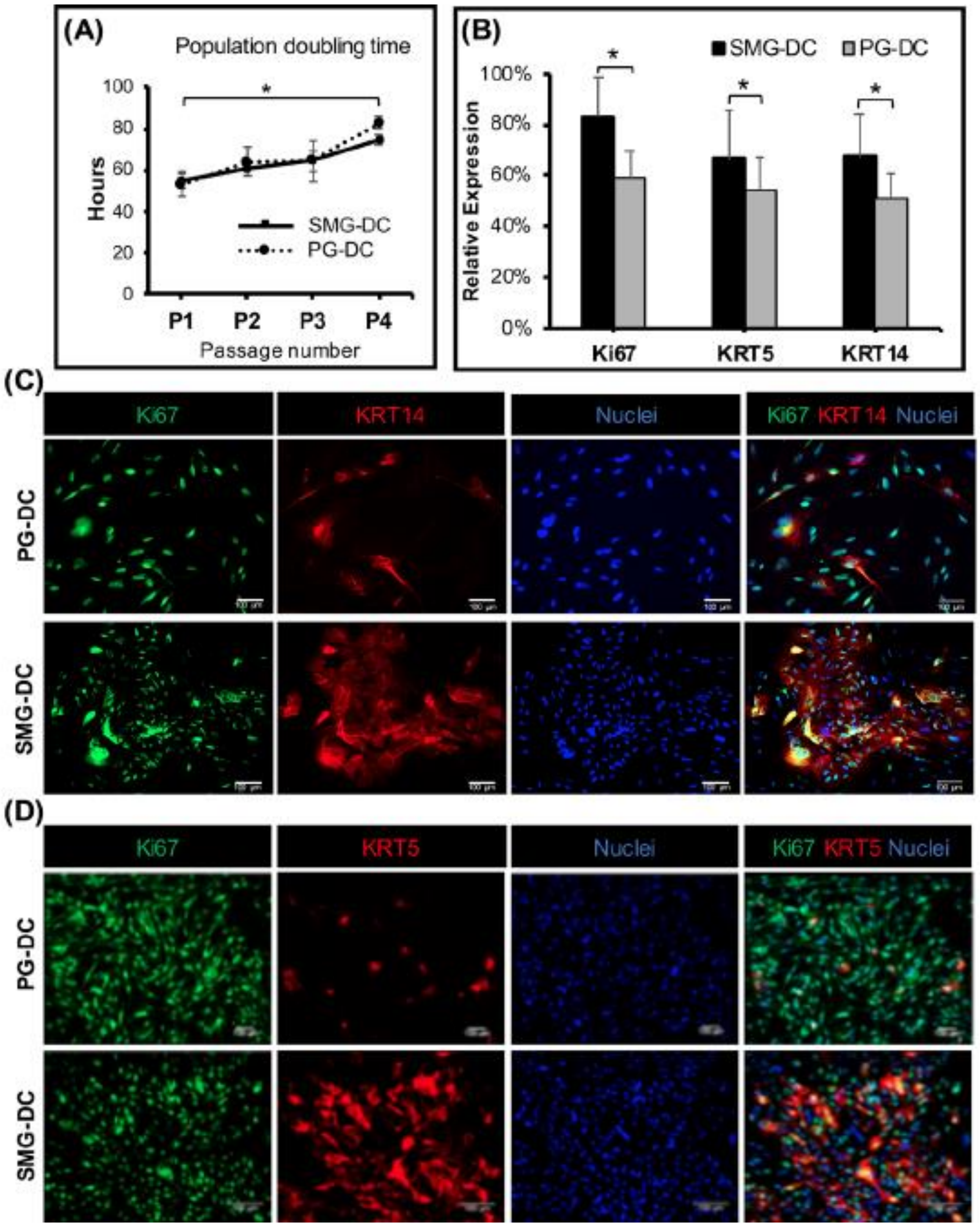


**Figure S2.** Cellular expansion through passaging and proliferation of epithelial progenitors in non-confluent undifferentiated SMG-DC and PG-DC cultures. **(A)** Cellular expansion was assessed by population doubling time (PDT) from passage 1 through 4 (P1–P4) of cell cultures. No significant difference was found between the first 3 passages ($N = 3–5$). *$p < 0.05$ when comparing passage 1 to 4. There were no differences in the PDT between SMG and PG through passage 1 to 4. **(B)** Quantification of Ki67+, KRT14+, KRT5+ cell subpopulations in SMG-DC and PG-DC. The y-axis represents protein expression relative to total nuclear/cell counts. *$p < 0.05$ when comparing SMG-DC with PG-DC. SMG-DC: submandibular gland-derived cells. **(C)** Abundant expression of pro-mitotic proliferative markers (Ki67) and epithelial stem/progenitor cells (KRT14) in SMG-DC and PG-DC after immunofluorescence staining at first subculture. Scale bar: 100 µm. **(D)** Expression of pro-mitotic proliferative markers (Ki67) and epithelial stem/progenitor cells (KRT5) in SMG-DC and PG-DC after immunofluorescence staining at first subculture. Scale bar: 100 µm. PG-DC: parotid gland-derived cells. SMG-DC: submandibular gland-derived cells.

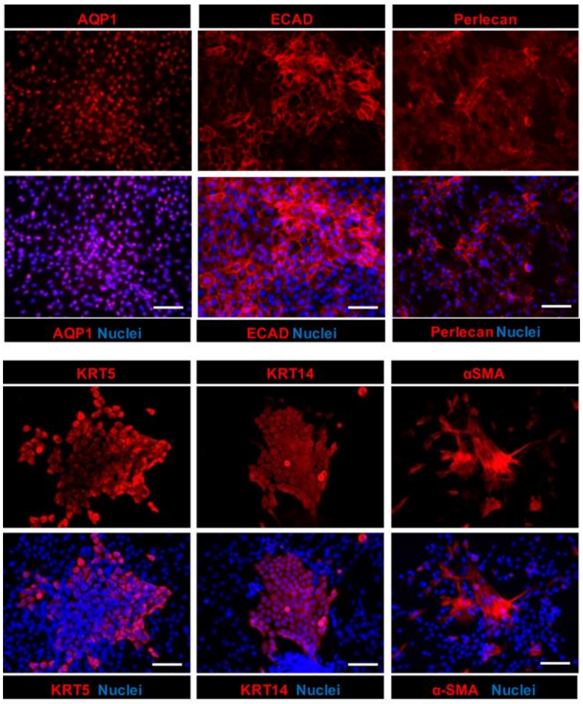


**Figure S3.** Expression of SG-specific acinar and ductal epithelial, myoepithelial and basement membrane markers after SG differentiation. Fluorescence microscopy images with z-stack maximum intensity projections are shown from immunohistochemistry for Aquaporin 1 (AQP1), E-cadherin (ECAD), KRT5, KRT14, Smooth muscle actin (αSMA) epithelial markers and counterstained with a nuclear dye. Perlecan was used to identify the basement membrane. Scale bar: 100 μm.

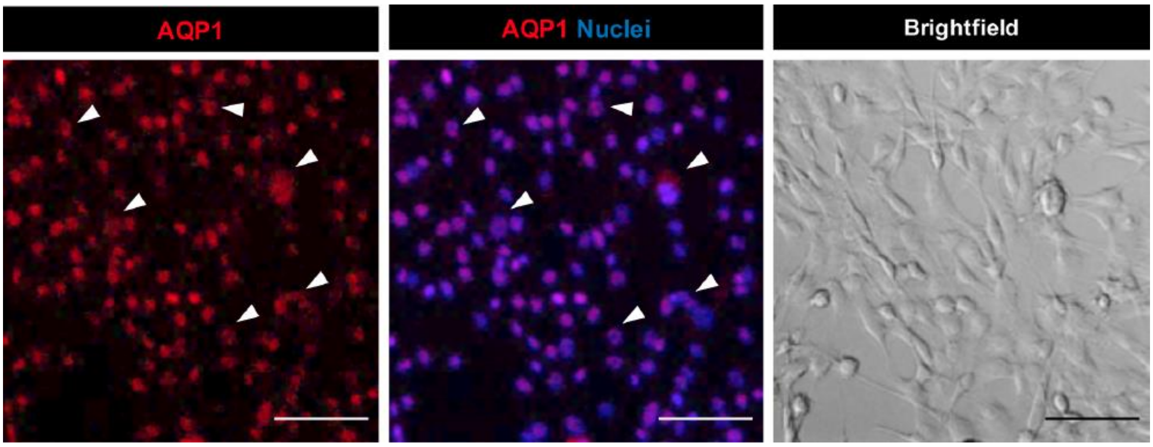


**Figure S4.** AQP1 water channels were not precisely located at the apical membrane after SG differentiation. Fluorescence microscopy images with z-stack maximum intensity projections are shown from immunohistochemistry for AQP1 at higher magnification, and counterstained with a nuclear dye. AQP1 appeared punctate around and overlapping the nuclei (white arrowheads) since this is a projection of multiple stacks. Scale bar: 100 μm.

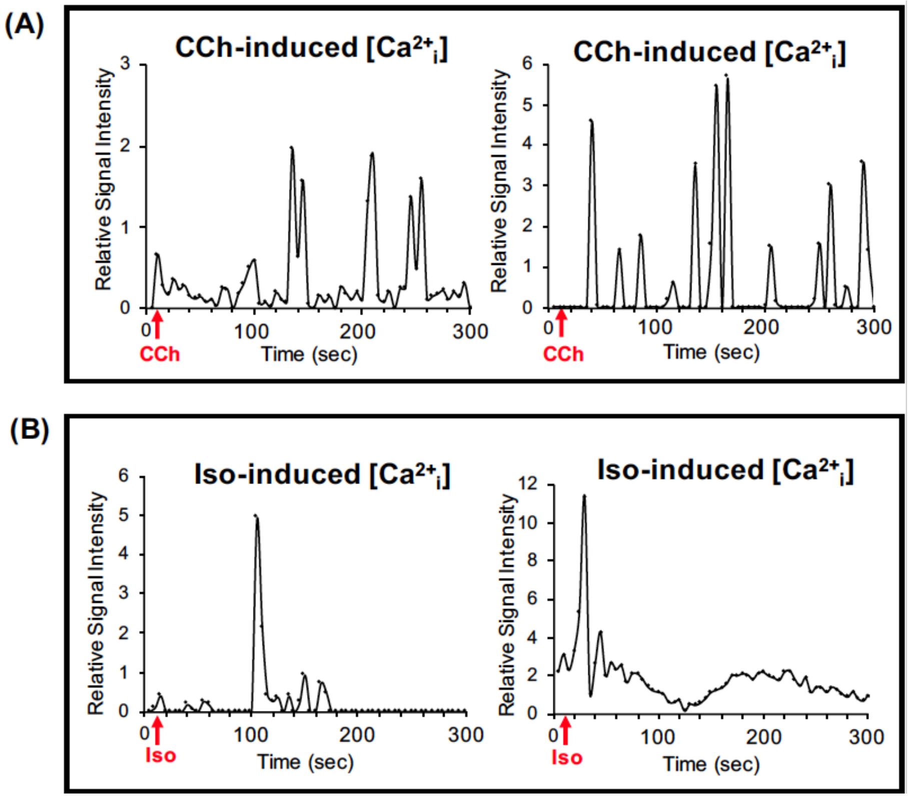


**Figure S5.** Intracellular $Ca^{2+}$ influx tracking in differentiated SG cells upon cholinergic and adrenergic stimulation. (**A**,**B**) Intracellular calcium influx in differentiated SG cells after (**A**) cholinergic stimulation with Carbachol (CCh) and (**B**) adrenergic stimulation with Isoproterenol (Iso).
